# Correlation Between eHealth Literacy and Health Literacy Using the eHealth Literacy Scale and Real-Life Experiences in the Health Sector as a Proxy Measure of Functional Health Literacy: Cross-Sectional Web-Based Survey

**DOI:** 10.2196/jmir.9401

**Published:** 2018-10-31

**Authors:** Pietro Del Giudice, Giulia Bravo, Marco Poletto, Anna De Odorico, Alessandro Conte, Laura Brunelli, Luca Arnoldo, Silvio Brusaferro

**Affiliations:** 1 Department of Medicine University of Udine Udine Italy

**Keywords:** eHealth literacy, health literacy, effect size, eHEALS, lily model

## Abstract

**Background:**

The eHealth Literacy Scale (eHEALS) is a tool for the self-assessment of perceived comfort and skills in using the internet as a source for health-related information. Although evidence exists of the reliability and construct and structural validity of the scale, there is a lack of evidence in relation to what is proposed by Norman and Skinner in their theoretical lily model of eHealth literacy; in particular it is not clear whether having a higher level of health literacy can positively influence electronic health (eHealth) literacy as measured by the eHEALS.

**Objective:**

Our study aim was to assess whether real-life experiences from studying or working in the health field, as a proxy of higher functional health literacy, correlate with self-referred eHealth literacy as measured by the eHEALS.

**Methods:**

A Web-based survey was conducted among adults living in Northeast Italy using an Italian version of the eHEALS (IT-eHEALS). In order to be able to measure the effect of higher functional health literacy on eHealth literacy, we divided our sample into two groups, respectively characterized by studying or working experience in the health sector and by lack thereof. Mean differences between eHEALS were calculated using t test and effect size evaluated using Cohen d. To ensure the validity of the IT-eHEALS, we evaluated its psychometric properties (internal consistency and dimensionality) and construct validity (by evaluating its correlation with respondents age, gender, educational attainment, self-rated health, use of internet for health-related purposes, and working status).

**Results:**

A total of 868 respondents that completed the IT-eHEALS were included for analysis, of which 259 had working or studying experience in the health field. Mean (SD) eHEALS total score was 28.2 (6.2) for the whole sample, with statistically significant differences (*P*<.001) between the two groups, with the higher health literate group scoring significantly better (31.9 (5.9) vs 26.7 (5.6), respectively), with a standardized mean difference (Cohen d) of 0.9. Interestingly, we found a weak, yet significant, correlation between eHealth literacy and respondent characteristics for the higher health literate group only, as measured by positive Spearman correlation coefficients for age (0.11, *P*=.001), educational attainment (0.19, *P*=.002) and self-rated health (0.14, *P*=.024). Also, in line with current literature, correlation of eHEALS score with frequency of internet use for health-related purposes was significant for both groups (0.32, *P*<.001 and 0.15, *P*<.001 for higher and lower health literacy group, respectively). In our study we could not find any difference related to gender, while a significant difference for working status was only present when considering the sample as a whole (*P*=.03).

**Conclusions:**

Our study demonstrates a sizeable effect of higher levels of functional health literacy on the eHEALS score, corroborating what was initially proposed by Norman and Skinner in the lily model of eHealth literacy.

## Introduction

### Health Information and the Internet

Use of the internet for health-related purposes poses a particularly important challenge, as it has been shown that wrong or incomplete information available on the internet may have negative consequences for the user—including on doctor-patient relationships, participation in prevention and screening programs, or adherence to medical treatment [1]. Today, the availability and accessibility of quality health-related internet information is still an issue, and agreement on a specified set of quality standards for health websites has recently been proposed as a new public health priority [2]. The problem of providing quality health-related information has become even more complex in the current Web 2.0 environment, as the search strategy for relevant information depends not only on the searcher’s ability, but also on the influence of intermediators and apomediators, with the latter effectively pushing the search towards or away from relevant items [3].

### eHealth Literacy and the eHEALS

In 2006, after three years of experimentation in a teenage health promotion program, Norman and Skinner developed the concept of eHealth literacy, drawing from the increasingly popular concept of health literacy. In the same year, the authors proposed both a theoretical model [4] and a tool to measure the new construct [5]. In their view, eHealth literacy was defined as “the ability to seek, find, understand, and appraise health information from electronic sources and apply the knowledge gained to addressing or solving a health problem.” The proposed model, called “lily model”, described eHealth literacy as the interplay of six core skills or literacies (traditional literacy, health literacy, information literacy, scientific literacy, media literacy, and computer literacy). The measurement tool, called eHealth literacy scale (eHEALS), was based on the principles of the social cognitive theory and self-efficacy theory, using Likert scales for self-assessed, subjective responses, so that measures should be considered as precursors of behavior change and skill development [6]. In this sense the eHEALS can be considered a measure of subjective, self-assessed eHealth literacy.

To further improve this first attempt to measure eHealth literacy, recent years have seen the development and validation of more comprehensive, and thus complex, eHealth literacy evaluation tools [7-9]. It should be noted, though, that despite all the possible issues coming from the simple eight-item structure of the eHEALS, its simplicity is also a strength of the tool. In fact, at the time of its development, the eHEALS was explicitly conceived so that it would have been easy to administer, taking into consideration the expressed needs of health professionals who said that they wouldn’t use a long instrument in their practice [10]. This “strength in simplicity” facilitated the adoption of the eHEALS, and its widespread use has been highlighted in the findings of several literature reviews. In 2012 Collins and colleagues reviewed the use of health literacy screening tools in eHealth applications [11] and found that the eHEALS was the most used for the purpose of developing a computer-based instrument to screen individuals accessing eHealth applications, alone or in combination with other screening tools for health literacy. In a 2015 review of existing tools to measure eHealth literacy and their use by Karnoe and Kayser [12], the authors found that, out of eight different tools for measuring eHealth literacy, only the eHEALS had been used in studies other than the one it was originally published in. The same authors argue that the eHEALS, while easy to administer, provides a measure that is not able to pinpoint whether inadequate eHealth literacy is a result of insufficient health literacy, digital literacy, or a combination hereof. In other words, it is still not clear whether a higher level of self-referred eHealth literacy (using the eHEALS), is correctly due to differences in levels of functional health literacy or is just a result of high levels of perceived self-efficacy.

### Aim of Current Study

The sheer amount of unchecked health-related information on the internet can be seen either as a limit or as a resource by different respondents with different skills and experiences in the health field. Considering the subjective, self-referred nature of the eHEALS, one possibility could be that people with less knowledge in the health field would trust the information more as they would be less able to discern the real quality of their internet search findings, scoring higher in the scale. The aim of our study is to test the lily model, by assessing whether and to what extent differences in health literacy levels account for variations in the eHEALS score. To our knowledge, no prior study using the eHEALS explored whether the scale behaves as intended in populations with different sets of core skills or literacies as described in the lily model, in our case based on differences in health literacy levels. In their description of the lily model of eHealth literacy, Norman and Skinner use the definition of health literacy given by the American Medical Association [13], which can be arguably referred to as a basic “functional” level of health literacy [14]. There are several possible measures of functional health literacy using measurement tools that are grounded on different theories. Yet, functional measures of health literacy have been shown to correlate with studying or working experiences in the health field [15], with physicians, researcher in the health field, and nurses showing higher health literacy levels in comparison with the general population. Therefore, we chose to recruit a large sample of respondents, divided into two groups, by asking them whether they had real-life experiences in studying or working in the health sector (eg, physicians, nurses, health alliance professionals). By doing so, we were able to compare a highly health-literate group with the rest of the general population.

## Methods

### Survey Design and Administration

In order to test our hypothesis, during November and December 2016 a Web-based survey was conducted by contacting people using two different recruitment strategies. Recruitment was performed using: (a) the mailing list of the student body (undergraduate and post-graduate) from the University of Udine (obtained with permission from the University), and (b) Facebook contacts of the public health research team members, who were then asked via Facebook to further disseminate the survey to their contacts. Decision to participate in the survey was voluntary and no incentives were offered to respondents. The survey was first pretested for usability and functionality by the members of the research team. The survey was administered using the software SurveyMonkey. All participants were asked to read and approve an informed consent form telling them that the study was managed by the University of Udine and that the survey would require approximately 15 minutes. As the survey did not collect any data that could be directly linked to participants’ sensitive data or information that could potentially affect their health, no approval by the Ethical Committee was deemed required under Italian legislation.

### Measures

Collected measures covered socio-demographic characteristics (gender, age, highest educational level attained, working status), self-perceived health status, internet health-related behaviors (use for health-related search and frequency), working or studying experiences in the health sector, and an Italian adaptation of the eHEALS scale. Age was collected as a discrete variable, in number of years. Educational attainment was first collected using an 8-item scale, later aggregated into a 3-item scale in line with the aggregation methodology used by Eurostat in relation to International Standard Classification of Education levels [16]. The final set of education levels used for analysis were: (low) 8th grade or lower, (middle) 9-13th grade, (high) university degree or higher. Working status was collected asking participants whether they were currently working, studying, or neither working nor studying (classified as “other”). Self-rated health was collected using a 5-item Likert scale, ranging from “very bad” to “excellent,” with the midpoint rated as “good.” Health-related internet use was measured asking the frequency of internet use for health-related purposes (using a 5-item Likert scale ranging from “not more than 5-6 times a year” to “several times a week”). To differentiate for real-life experiences in the health sector, participants were asked whether they had experiences in studying or working in the health sector using a yes/no question. Regarding the Italian version of the eHEALS, we were unable to retrieve a previously reported version of the tool (I-eHEALS) presented in a conference abstract by De Caro et al [17] (via request to the corresponding author), and a new Italian translation of the 8 eHEALS items was produced by the research team (IT-eHEALS). The translation process was carried out following established good practices [18]: the original English tool was initially distributed among the research team, producing a first set of translations that were later merged into a single draft version. The draft of the Italian instrument was then retranslated into English by an interpreter and reviewed by the research team for correctness. Translated items were pretested for comprehensibility on a small sample of Italian adults (N=24) and items were adjusted accordingly. Like the original version of the test, the IT-eHEALS is composed of 8 items measured with a 5-point Likert scale. For every respondent of the sample that completed all 8 IT-eHEALS items, the total score ranges from 8 to 40 (calculated by adding up the single items’ scores), with a higher score indicating a higher self-referred eHealth literacy.

### Statistical Analysis

#### Sample Selection and Descriptive Analysis

To test our hypothesis, we selected the subsample of respondents who completed all of the 8 IT-eHEALS items. All collected data were screened to search for missing values or for any incorrect data inclusion. When not plausible, records were excluded from the analysis upon discussion among the research team. Then, the sample was divided into two groups based on having experiences of studying or working in the health sector or not. In this paper, we will refer to the group currently studying or working in the health sector as EHS+, and to the other as EHS-. Descriptive statistics (frequency, percentage, mean [SD]) were calculated for socio-demographic variables (gender, age, educational attainment, and working status), self-rated health, and internet health-related behaviors for all groups. A comparative analysis using Wilcoxon-Mann Whitney test and Chi-square (or Fisher Exact) Test, respectively for continuous and categorical variables, was conducted to detect statistically significant group differences (*P*<.05).

#### IT-eHEALS Scale Validity

Since we used a newly developed and adapted Italian version of the eHEALS (IT-eHEALS), we also assessed the scale by examining its psychometric properties and construct validity. Psychometric properties were examined by measuring internal consistency (Cronbach alpha) and conducting a principal component analysis to assess the dimensionality of the scale. Construct validity was assessed using a hypothesis testing approach. Based on prior studies, it was hypothesized that participants who (a) are younger [19], (b) use the internet for health-related purpose more frequently [20], (c) have a better self-rated health [17,21], and (d) have higher educational attainment [19], would have higher self-referred eHealth literacy scores. Spearman rho index was used to assess correlations between IT-eHEALS total score and (a) age, (b) internet use, (c) self-rated health and (d) educational level in the two groups of IT-eHEALS respondents. Also, we used *t* test and analysis of variance (ANOVA) to evaluate the difference in IT-eHEALS scores for gender and working status, respectively.

#### Relation Between Health and eHealth literacy

Finally, differences between eHEALS means and SDs in the EHS+ and EHS- groups were calculated using *t* test, and effect size was evaluated using Cohen *d*. Analysis was conducted using SAS software version 9.4 for Windows (SAS Institute Inc, Cary, NC, USA).

## Results

### Socio-Demographic Characteristics

In total, the two internet surveys led to the recruitment of 1136, of which 868 completed all eight IT-eHEALS items, leading to a final sample of 868 respondents that were included for analysis. Table 1 shows the socio-demographic characteristics of the whole sample and differences between the EHS+ and EHS- groups. The two groups differ significantly (P<.001) in relation to working status and frequency of internet use for health-related purposes. In the EHS+ group, most of the respondents are working (139/259, 53.7%), while in EHS- the majority are studying (303/609, 49.7%). Regarding internet use for health-related purposes, 27.4% (71/259) of EHS+ respondents use the internet more than once a week, while only 5.42% (33/609) of the EHS- respondents do so, suggesting differences in health-related internet behaviors between the two groups. Also, respondents in the EHS+ group are significantly older, with a mean age of 31.5±12.1 years vs 28.7±9.7 years for the EHS- group (*P*=.008).

### Validity of the IT-eHEALS Scale

IT-eHEALS showed a high degree of internal consistency with a Cronbach alpha of .90, with slight, negligible differences between the two groups (.87 in EHS-, .91 in EHS+). Principal Component Analysis in the whole sample confirmed the unidimensionality of the scale (eigenvalue=4.9 with 61.1% of variance explained). All IT-eHEALS items show high loadings on the first component (ranging from 0.68 to 0.83). Table 2 shows Spearman correlation coefficients with age and educational attainment. Correlation coefficients of total mean scores on the IT-eHEALS with selected variables are significant but low, with the exception of age, educational attainment, and self-rated health in EHS-. The correlation with frequency of internet use for health-related purposes was significant in both groups.

We did not find any difference in relation to gender. When assessing the whole sample, there was a significant difference for working status (*P*=.03) that was not present when considering EHS+ and EHS- separately in both groups.

**Table 1:** Descriptive and comparative analysis of study sample.

Variable		Whole sample (N=868), n (%)	EHS+^a^ (N=259), n (%)	EHS-^b^ (N=609), n (%)	*P* value^c^
**Gender**					. 85
	Male	231 (26.6)	70 (27.0)	161 (26.4)	
	Female	637 (73.4)	189 (73.0)	448 (73.6)	
**Educational attainment**					.057
	Low	22 (2.5)	5 (1.9)	17 (2.8)	
	Middle	457 (52.7)	121 (46.7)	336 (55.2)	
	High	383 (44.1)	129 (49.8)	254 (41.7)	
	No response	6 (0.7)	4 (1.6)	2 (0.3)	
**Working status**					<.001
	Working	391 (45.1)	139 (53.7)	252 (41.4)	
	Studying	416 (47.1)	113 (43.6)	303 (49.7)	
	Other	61 (7.0)	7 (2.7)	54 (8.9)	
**Self-rated health**					.27
	Very bad	6 (0.7)	1 (0.4)	5 (0.8)	
	Poor	62 (7.1)	21 (8.1)	41 (6.7)	
	Good	455 (52.4)	123 (47.5)	332 (54.5)	
	Very good	281 (32.4)	90 (34.7)	191 (31.4)	
	Excellent	64 (7.4)	24 (9.3)	40 (6.6)	
**Frequency of internet use for health-related purposes**					<.001
	No more than 5-6 times/year	282 (32.5)	62 (23.9)	220 (36.1)	
	No more than 2-3 times/year	135 (15.5)	31 (12.0)	104 (17.1)	
	Once a month	238 (27.4)	58 (22.4)	180 (29.6)	
	Once a week	109 (12.6)	37 (14.3)	72 (11.8)	
	Several times a week	104 (12.0)	71 (27.4)	33 (5.4)	

^a^EHS+: Group with studying or working experiences in the health sector.

^b^EHS-: Group without studying or working experiences in the health sector.

^c^*P* values are calculated for mean differences between groups EHS+ and EHS-.

**Table 2:** Spearman correlations between eHealth Literacy Scale total score for selected variables.

Variable	Whole sample		EHS+^a^		EHS-^b^	
	Spearman correlation coefficient	*P* value	Spearman correlation coefficient	*P* value	Spearman correlation coefficient	*P* value
Age	0.11	.002	0.22	.001	0.02	.65
Educational attainment	0.11	.001	0.19	.002	0.06	.13
Self-rated health	0.07	.038	0.14	.024	0.02	.70
Frequency of internet use for health	0.28	<.001	0.32	<.001	0.15	<.001

^a^EHS+: Group with studying or working experiences in the health sector.

^b^EHS-: Group without studying or working experiences in the health sector.

**Table 3:** Italian version of eHealth Literacy Scale (eHEALS) items and total score statistics.

eHEALS score	Whole sample (N=868), mean (SD)	EHS+^a^ (N=259), mean (SD)	EHS-^b^ (N=609), mean (SD)	*P* value^c^
Item 1	3.8 (0.9)	4.2 (0.8)	3.6 (0.8)	<.001
Item 2	3.5 (0.9)	3.9 (1.0)	3.4 (0.9)	<.001
Item 3	3.6 (1.0)	4.0 (0.9)	3.4 (0.9)	<.001
Item 4	3.7 (0.9)	4.1 (0.9)	3.5 (0.9)	<.001
Item 5	3.7 (0.9)	4.1 (0.9)	3.6 (0.9)	<.001
Item 6	3.5 (1.2)	4.2 (1.0)	3.2 (1.1)	<.001
Item 7	3.8 (1.0)	4.2 (0.8)	3.6 (1.0)	<.001
Item 8	2.7 (1.2)	3.2 (1.2)	2.4 (1.1)	<.001

^a^EHS+: Group with studying or working experiences in the health sector.

^b^EHS-: Group without studying or working experiences in the health sector.

^c^*P* values are calculated for mean differences between groups EHS+ and EHS-.

### Health Literacy and the eHEALS

Table 3 shows the mean (SD) item score and the statistical significance of the difference between the EHS+ and EHS- groups (see Multimedia Appendix 1 for item descriptions). Considering the whole sample of IT-eHEALS respondents, mean values for items range from 3.8 (item 1) to 2.7 (item 8). Differences between the two groups were significant for all IT-eHEALS items (*P*<.001), with the mean (SD) total score significantly higher in EHS+ compared to EHS- (31.9 [5.9] vs 26.7 [5.6], *P*<.001). The standardized mean difference (Cohen *d*) was 0.9, demonstrating a sizeable effect of higher levels of functional health literacy on the eHEALS score.

## Discussion

### Study Findings

#### Correlation Between Health Literacy and eHealth Literacy

In our study we were able to demonstrate that real-life working or studying experiences in the health sector, as a proxy of higher levels of health literacy, positively correlate with self-referred eHealth literacy as measured by the eHEALS. This finding is in line with the original lily model of eHealth literacy proposed by Norman and Skinner, where eHealth literacy is described as the interconnection of different core skills, including health literacy. Our findings emphasize that there are different factors other than internet and computer skills that can lead to different results when measuring eHealth literacy.

#### Psychometric Characteristics and Construct Validity of the eHEALS

Regarding the validity of the IT-eHEALS in the Italian population, we found high internal consistency, as shown by the Cronbach alpha and the inter-item correlation analysis, with comparable results with other translation of the eHEALS [19-26]. Our principal component analysis shows that the IT-eHEALS can be better explained by a single component structure, supporting its unidimensionality. While authors of two past studies using the eHEALS argued that the scale could have been multidimensional [24,27], our results are in line with other studies that confirmed the unidimensional nature of the scale, which allow for the calculation of a total mean score of all the eHEALS items [20,28,29]. Regarding the construct validity of the eHEALS, interpretation of our findings should be taken cautiously due to possible bias introduced by the sampling technique and keeping in mind that the sample was composed of young adults aged 20-30. Also, as already noted by Diviani et al [20], most of eHEALS validation studies have been conducted among specific populations, with different results showing no consistent association of eHEALS scores with the personal characteristics of the respondents, such as gender, education, or age. In fact, while some studies found significant correlation of eHealth literacy levels with age [19,26], education [19], gender [26] and self-rated health [17,21], other studies found no correlation for the same variables. In particular, several other studies found no correlation between the eHEALS and gender [19-21], age [20], and education [20]. Our study findings show that the IT-eHEALS have a weak, positive correlation with age, educational attainment, and self-rated health. It must be noted that, interestingly, when considering our two subsamples separately, these correlations show a level of significance only in EHS+, while this is not true for EHS-, suggesting a correlation between different levels of functional health literacy and self-referred eHealth literacy. Regarding gender, we found no correlation with the eHEALS score, a result that is comparable with other studies involving a similar young and highly educated population [20,21]. Also, in line with similar studies [20], we found a weak level of correlation with the frequency of internet use for health-related purposes in all groups. Overall, these results suggest that the eHEALS should be considered a valid tool that can be used to assess the perceived comfort and skills in using the internet for health-related purposes.

### Study Limitations

Our study has some limitations that should be acknowledged.

#### Sample Composition

A first limitation of our study lies in the recruitment strategy used, which led to a study sample which is composed by respondents who are mostly young and highly educated, and therefore could not be considered representative of the adult Italian population, limiting the generalizability of our findings. While the English version of the scale has been applied in a variety of samples, most of the validating studies of the eHEALS in other languages have only been conducted among specific populations. Regarding gender, our sample has an overrepresentation of female respondents, so that our results shall be taken cautiously when trying to generalize to the general adult population. Also, it should be noted that the use of Facebook in our recruitment strategy made it impossible to assess number and characteristic of nonrespondents, an important limitation that should also be considered when interpreting results. While these are common shortcoming of similar validation studies, we believe that its composition characteristics (higher education level, younger age) are somewhat representative of the most active population of health information seekers in the internet, as reported by the latest 2017 EU Digital Scoreboard statistics for Italy about health information seeking in the general population (see Multimedia Appendix 2). Moreover, our study population was sufficient to address our aim, namely the recruitment of a sample large enough to be divided into two comparable groups characterized by study or work experiences in the health sector. In relation to this point, we are also aware that the two groups were not equally distributed for some of the socio-demographic and health-related internet behavior factors; since our methodology did not allow us to select our sample composition beforehand, we cannot be sure whether group differences are an effect of the selected variable for group inclusion (in our case people with experiences in the health sector having different baseline characteristics compared to the general population for age, working status and use of internet for health-related purposes), or whether there are other reasons for these differences that are not due to the recruitment techniques we used.

#### Measures

Another limitation of our study lies in the fact that we only included one measure of internet health-related behavior, as comparing different measures was outside the original scope of the study. While it should be acknowledged that this measure has not been previously validated, our results suggest that the two groups may indeed be different in terms of internet health-related behaviors, yet these should be further explored with a larger number of measures before reaching definitive conclusions on the health literacy role in explaining behavioral differences in this field. Also, we did not include any validated measures of either subjective or objective health literacy, which could have been used to quantitatively assess different levels of health literacy. Instead, we asked for real-life experiences in the health field as a proxy, which have been showed to correlate only with objective health literacy tests [15]. Our results show that there is a correlation between these experiences and the eHEALS, yet we suggest that future studies also include other validated measures of health literacy to better correct results and to explore the correlation of the eHEALS with both subjective and objective measures of health literacy. Another limitation of the present study is the lack of an objective measure of eHealth literacy skill, making it unclear whether the differences between groups in health-related internet behaviors could also be related to actual, objective eHealth literacy skills. At the moment, there are mixed results regarding the correlation between eHEALS and objective measures of competencies on health-related internet use: using different measures of eHealth literacy objective competencies, Neter and Brainin found moderate correlation [30], while van der Vaart et al found no correlation [23]. This is also common to other measures used in the field of health literacy and is probably due to the subjective nature of the tools used [31], and even in the presence of a moderate correlation, Neter and Brainin recommend assessing the two constructs separately [30]. As these authors are providing the methodological base for more objective eHealth literacy measures, we also encourage future studies to include measures based on these methodologies [30,32]. This would not only lead to a better comprehension of the relation between subjective and objective measures, but it would also contribute to the possibility of expanding the item bank of objective measures for future studies, with possible use of advanced theories for test development such as Item Response Theory or the Rasch model [20,31].

#### eHEALS Version

It must be noted that after our study was conducted, a validation study of another Italian version of the eHEALS (I-eHEALS) was published by Diviani et al, using a sample population of Italian-speaking Swiss respondents [20]. As we used a different Italian translation of the eHEALS, it remains unclear whether results could be comparable to their results. While there are minor differences in the phrasing of the items, our scale shows good internal consistency and construct validity. For this reason, we believe that the two currently available Italian translations of the eHEALS (I-eHEALS and IT-eHEALS) can both be considered valid and, in our opinion, can be used interchangeably (see Multimedia Appendix 1 for eHealth Literacy Scale Italian versions).

### Conclusions

This study demonstrates that, as proposed in the lily model of eHealth literacy, eHEALS scale results are affected by a higher level of health literacy, measured via real-life experiences in the field of health as a proxy. We believe that this is an original result, which could be relevant in the current stage of scientific discussion regarding the use of the eHEALS and further advancements in measuring eHealth literacy. Despite its several limitations, and in absence of simple, easy-to-administer measurement tools, the eHEALS can still be considered a valid tool to assess self-perceived comfort and skills in using the internet for health-related purposes. It should still be used for comparison in the elaboration of new eHealth literacy measures, which should be designed including new items and different subscales in order to be able to capture all the proposed “literacies” of the construct [4]. For these reasons, we believe that the absence of correlation of the eHEALS with objectively measured internet related skills as found by different authors does not undermine the validity and the usability of the scale per se, and that the eHEALS can still be applied in clinical and health promotion activities, for example to identify different needs for the participants to an eHealth intervention or to evaluate intervention results.

## Appendix

Multimedia Appendix 1 eHealth Literacy Scale Italian versions.

Multimedia Appendix 2 Internet health-information seeking behavior in the Italian adult population.

## Abbreviations

ANOVA: analysis of variance
eHEALS: eHealth Literacy Scale
eHealth: electornic health
EHS+: Group with studying or working experiences in the health sector
EHS-: Group without studying or working experiences in the health sector
I-eHEALS: Swiss-Italian version of the eHealth Literacy scale
IT-eHEALS: Italian version of eHealth Literacy Scale
