# Supplementary material for: Correlation Between eHealth Literacy and Health Literacy Using the eHealth Literacy Scale and Real-Life Experiences in the Health Sector as a Proxy Measure of Functional Health Literacy: Cross-Sectional Web-Based Survey
Source: J Med Internet Res. 2018 Oct 31;20(10):e281. doi: 10.2196/jmir.9401 (PMC6246968; doi:10.2196/jmir.9401)
Supplement: Multimedia Appendix 1 [file jmir_v20i10e281_app1.pdf]

Multimedia Appendix 2. eHealth Literacy Scale Italian versions (IT-eHEALS and I-eHEALS).

Item	Original eHEALS[a]	IT-eHEALS[b]	I-eHEALS[b,c]
1	I know how to find helpful health resources on the Internet	So come trovare su internet informazioni utili sulla salute	So come trovare informazioni utili riguardanti la salute in Internet
2	I know how to use the Internet to answer my health questions	So come usare internet per rispondere alle domande riguardo la mia salute	So come utilizzare Internet per trovare risposte alle mie domande sulla salute
3	I know what health resources are available on the Internet	So quali informazioni sulla salute sono disponibili in internet	So quali informazioni riguardanti la salute sono disponibili in Internet
4	I know where to find helpful health resources on the Internet	So dove trovare su internet informazioni utili sulla salute	So dove trovare informazioni utili riguardanti la salute in Internet
5	I know how to use the health information I find on the Internet to help me	So come usare le informazioni sulla salute che trovo su internet in modo che mi possano essere d'aiuto	So come utilizzare le informazioni riguardanti la salute trovate in Internet per aiutarmi
6	I have the skills I need to evaluate the health resources I find on the Internet	Ho le capacità che mi servono per valutare le informazioni sulla salute che trovo su internet	Ho le competenze necessarie per valutare le informazioni riguardanti la salute che trovo in Internet
7	I can tell high quality from low quality health resources on the Internet	Posso distinguere la bassa o alta qualità delle informazioni sulla salute che trovo su internet	Sono in grado di distinguere informazioni riguardanti la salute di alta qualità da quelle di bassa qualità presenti in Internet
8	I feel confident in using information from the Internet to make health decisions	Mi sento sicuro nell'usare informazioni che trovo su internet per prendere decisioni riguardo la mia salute	Mi sento sicuro/a nell'utilizzare informazioni ottenute da Internet per prendere decisioni riguardanti la salute

Notes:

[a] Source: Norman CD, Skinner HA. eHEALS: The eHealth Literacy Scale. J Med Internet Res 2006;8(4):e27. DOI: 10.2196/jmir.8.4.e27. PMID: 17213046. PMCID: PMC1794004.

[b] As in the English version, answers are on a 5-point Likert scale ranging from 1 = In completo disaccordo [Strongly disagree] to 5 = Completamente d'accordo [Strongly agree].

[c] Source: Diviani N, Dima AL, Schulz PJ. A Psychometric Analysis of the Italian Version of the eHealth Literacy Scale Using Item Response and Classical Test Theory Methods. J Med Internet Res 2017;19(4):e114 .DOI: 10.2196/jmir.6749. PMID: 28400356.PMCID: 5405289
