# Supplementary material for: Correlation Between eHealth Literacy and Health Literacy Using the eHealth Literacy Scale and Real-Life Experiences in the Health Sector as a Proxy Measure of Functional Health Literacy: Cross-Sectional Web-Based Survey
Source: J Med Internet Res. 2018 Oct 31;20(10):e281. doi: 10.2196/jmir.9401 (PMC6246968; doi:10.2196/jmir.9401)
Supplement: Multimedia Appendix 2 [file jmir_v20i10e281_app2.pdf]

Multimedia Appendix 1. Internet health-information seeking behavior in the Italian adult population. Data representation from the EU Digital Scoreboard of the EU Digital Single Market [a].

A) Internet health information seeking by age (six groups)

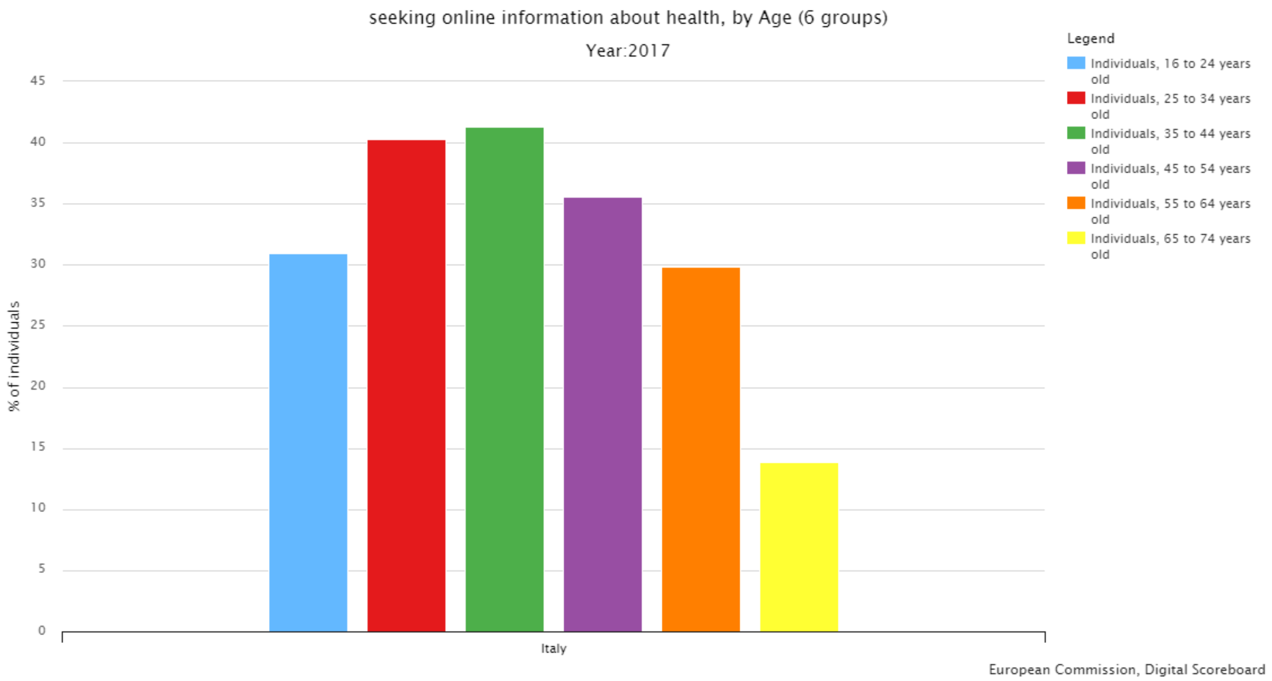

B) Health information seeking by educational attainment [b]

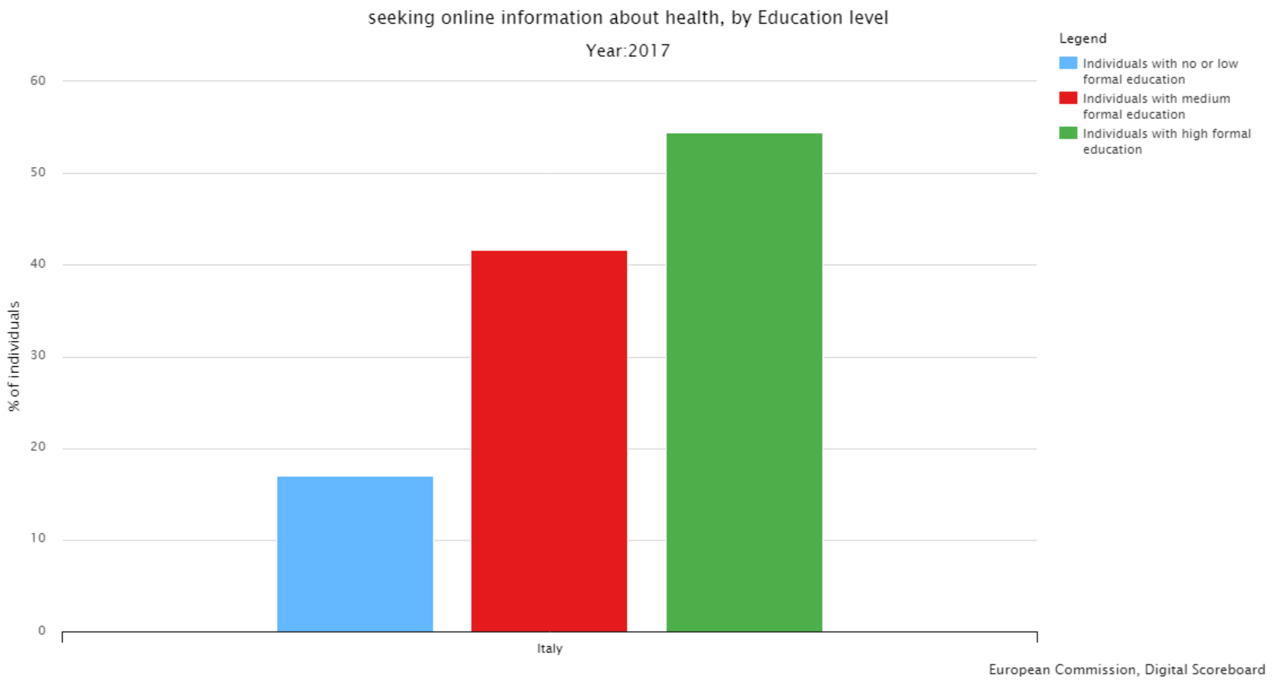

[a] Source: https://ec.europa.eu/digital-single-market/digital-scoreboard

[b] for interpreting levels of educational attainment, see also Eurostat. International Standard Classification of Education (ISCED). http://ec.europa.eu/eurostat/statistics-explained/index.php/International_Standard_Classification_of_Education_%28ISCED%29 Archived at: http://www.webcitation.org/70JugcUjL
